# On the phase consistency of apical organ of Corti vibrations

**DOI:** 10.1016/j.heares.2024.109137

**Published:** 2024-10-28

**Authors:** George W.S. Burwood, Tianying Ren, Alfred L. Nuttall, Anders Fridberger

**Affiliations:** a Oregon Hearing Research Center, Department of Otolaryngology–Head and Neck Surgery, Oregon Health & Science University, Portland, OR 97239, USA; b Department of Biomedical and Clinical Sciences, Linköping University, SE-581 83 Linköping, Sweden

**Keywords:** Low-frequency hearing, Cochlear mechanics, Optical coherence tomography, Traveling waves, Tonotopicity, Measurement precision, phase errors

## Abstract

Low-frequency hearing is critically important for speech and music perception. However, technical and anatomical limitations previously made it difficult to study the mechanics of the low-frequency parts of the cochlea, but this changed with the introduction of optical coherence tomography vibrometry. With this technique, sound-evoked vibration can be measured from the apex of a fully intact cochlea. Results of such measurements generated controversy because conventional traveling waves, the hallmark of which is longer group delay closer to the helicotrema, were absent within the apical 20% of the guinea pig cochlea (Burwood et al Science Advances 8:eabq2773, 2022). The validity of this result was questioned, primarily because group delays were calculated from phase values averaged across many points within the organ of Corti. Here we show that variations in phase across the organ of Corti are minor and does not affect the group delay significantly. We also assess the precision of phase measurements with optical coherence tomography. An artificial target with reflectivity similar to the organ of Corti was used. These measurements revealed that a commonly used commercial optical coherence tomography system produces half-cycle errors in 1–5 % of pixels, leading to a bimodal distribution of phase values. This problem can be easily addressed by using medians when computing averages, as was done by Burwood et al (2022). Hence, neither averaging across pixels nor technical factors can explain the apparent lack of conventional traveling waves at the apex of the guinea pig cochlea at low stimulus levels. The physiological mechanisms that operate at the apex apparently differ from other cochlear regions.

## 1. Introduction

Low-frequency sounds are critical for speech and music perception. This is evident from the fact that more than half the keys on a piano produces tones with fundamental frequency below 1 kHz, and from the fact that vowels can be recognized based on their first two formants, which also have frequencies below 1 kHz.

The apex of the cochlea, where low-frequency sounds are encoded, does not appear to function in the same manner as the extensively studied cochlear base. For instance, cat auditory nerve fibers innervating the apex show a downward frequency glide in response to click-like sounds, whereas an upward frequency glide is evident in fibers contacting basal-turn hair cells (Carney et al 1999). Similarly, van der Heijden and Joris (2006) studied cat auditory nerve fibers, concluding that “descriptions of the apex as a scaled version of the base will fail, because they miss qualitative differences between the apex and base”.

The human capacity for low-frequency hearing is however not shared by all animals. Mice only hear at high frequencies and among species with good low-frequency hearing, limited mechanical data are available from chinchillas and gerbils, so the bulk of our knowledge about low-frequency organ of Corti mechanics comes from guinea pigs.

Even in guinea pigs, it is challenging to study the part of the cochlea that encodes low-frequency sounds. A physiological difficulty is that opening of the cochlear capsule of the apex introduces artefacts (Ulfendahl et al 1991; Cooper and Rhode 1996; Ulfendahl et al 1996), but another difficulty is anatomical. Since the apex points in the anterior direction in commonly used laboratory animals, an extensive and often traumatic dissection is necessary to measure sound-evoked responses from the low-frequency parts of the cochlea *in vivo*.

Optical coherence tomography vibrometry (OCT; Wang and Nuttall 2010; Chen et al 2011; for reviews, see Olson and Strimbu 2020; Burwood et al 2019) made it possible to measure the sound-evoked response of the organ of Corti without opening the cochlear capsule, thereby eliminating concerns about artefacts. Additionally, the mirror arrangement described by Ramamoorthy et al (2016) enabled measurements with reduced surgical trauma. An initial study on a single location within the guinea pig apex revealed a vibration pattern that contradicted traditional models of cochlear function, as the basilar membrane was nearly motionless (Warren et al 2016). The OCT data in that study were however acquired at high stimulus levels and only a single cochlear location included.

Recio-Spinoso and Oghalai (2017) performed OCT measurements from three different apical sites in guinea pigs, demonstrating a compressive nonlinearity similar to the one evident at the base of the cochlea, but less pronounced. This was a contrast to some previous studies, which showed expansive nonlinearities (Zinn et al 2000; traditional vibrometry). Although not a conclusion entertained by the authors, it appeared to us that the pattern of frequency tuning shown in these studies was inconsistent with Greenwood’s commonly used cochlear place-frequency map (Greenwood 1990).

To address these inconsistencies, we performed a set of experiments to probe the frequency tuning of different sites within the apex. That study (Burwood et al 2022) showed that most sites within the apical 20 % of the guinea pig cochlea had nearly identical frequency tuning. The most apical measurement location was tuned to a higher frequency than the more basal ones, a sharp contrast with widely accepted models. Furthermore, at low stimulus levels there was no evidence for conventional traveling wave motion within the region examined, since all three measurement sites had similar group delay through much of the response region. Note that these results do not mean that we dispute the existence of traveling waves more generally. At issue is only whether a conventional traveling wave is present within the 20 % of the organ of Corti closest to the helicotrema.

Findings reported by Burwood et al (2022) were met with some skepticism. The averaging of cochlear vibrations across the width of the organ of Corti used by Burwood et al was considered problematic (Recio-Spinoso et al 2023), in contrast to the more commonly used single-point measurement technique. Here we address this concern through additional measurements and analyses.

These new analyses show that averaging phases across the organ of Corti is not a problem, because response phase is nearly constant across the organ of Corti at low stimulus levels. Furthermore, the minor differences that exist have no effect on the computed group delay.

To determine whether technical factors could contribute to the unexpected results in Burwood et al (2022), we performed calibration measurements using artificial targets with reflectivity similar to the apex of the cochlea. These measurements show that a commonly used commercial OCT system returns erroneous phase values in 1–5 % of pixels. This error can be avoided by using the median for averaging pixel phase values, as done by Burwood et al (2022).

## 2. Methods

The guinea pig data in the present paper is the same as in Burwood et al (2022). The purpose of the present paper is to address criticisms of that paper by performing new analyses, but we also include new measurements on artificial targets to address possible technical factors. These data are new and should be of interest for any researcher using OCT for cochlear vibration measurements.

All animal procedures were approved by the Institutional animal care and use committee at Oregon Health & Science University. Animals were deeply anesthetized with intramuscular injections of ketamine (40 mg/kg body weight) and xylazine (10 mg/kg for the first dose, 5 mg/kg for subsequent doses). Deep areflexia was maintained by injections repeated at approximately 45-min intervals, as guided by toe pinch.

To prepare the cochlea for recording of sound-evoked displacements, the soft tissue surrounding the auditory bulla was resected. A small opening in the bulla was then made using a scalpel blade, and a silver wire electrode placed in the round window niche. The electrode was secured to the bulla using dental cement and a loudspeaker placed in the ear canal. To ensure tight coupling between the speaker tube and the ear canal, grease was applied between the tube and the ear canal, and ear canal sound pressures were recorded repeatedly throughout the experiments using a Knowles FG-23329-P07 microphone positioned inside the speculum connecting the speakers to the ear canal. Any deviation in the calibration curve prompted repositioning of the speaker, whereafter the system was again calibrated until a satisfactory response was obtained.

Having calibrated the speaker system *in situ*, the compound action potential of the auditory nerve was measured at frequencies from 2 to 20 kHz. Since there are no physiological indicators that directly inform about the condition of the apical regions of the cochlea, we assumed that animals with normal auditory sensitivity in the 2 to 20 kHz frequency range also had normal apical function. Animals with compound action potential audiograms showing more than 10 dB loss were excluded from further study.

After ascertaining that a normal compound action potential audiogram was present, the opening in the bulla was gradually made wide enough to make insertion of a small mirror possible. The mirror was positioned inside the bulla and aimed at the apex; it obviates the need for extensive dissection of the jaw, enabling transverse sound-evoked displacements to be measured from the apex with much reduced surgical trauma (Ramamoorthy et al 2016).

**Fig. 1.**
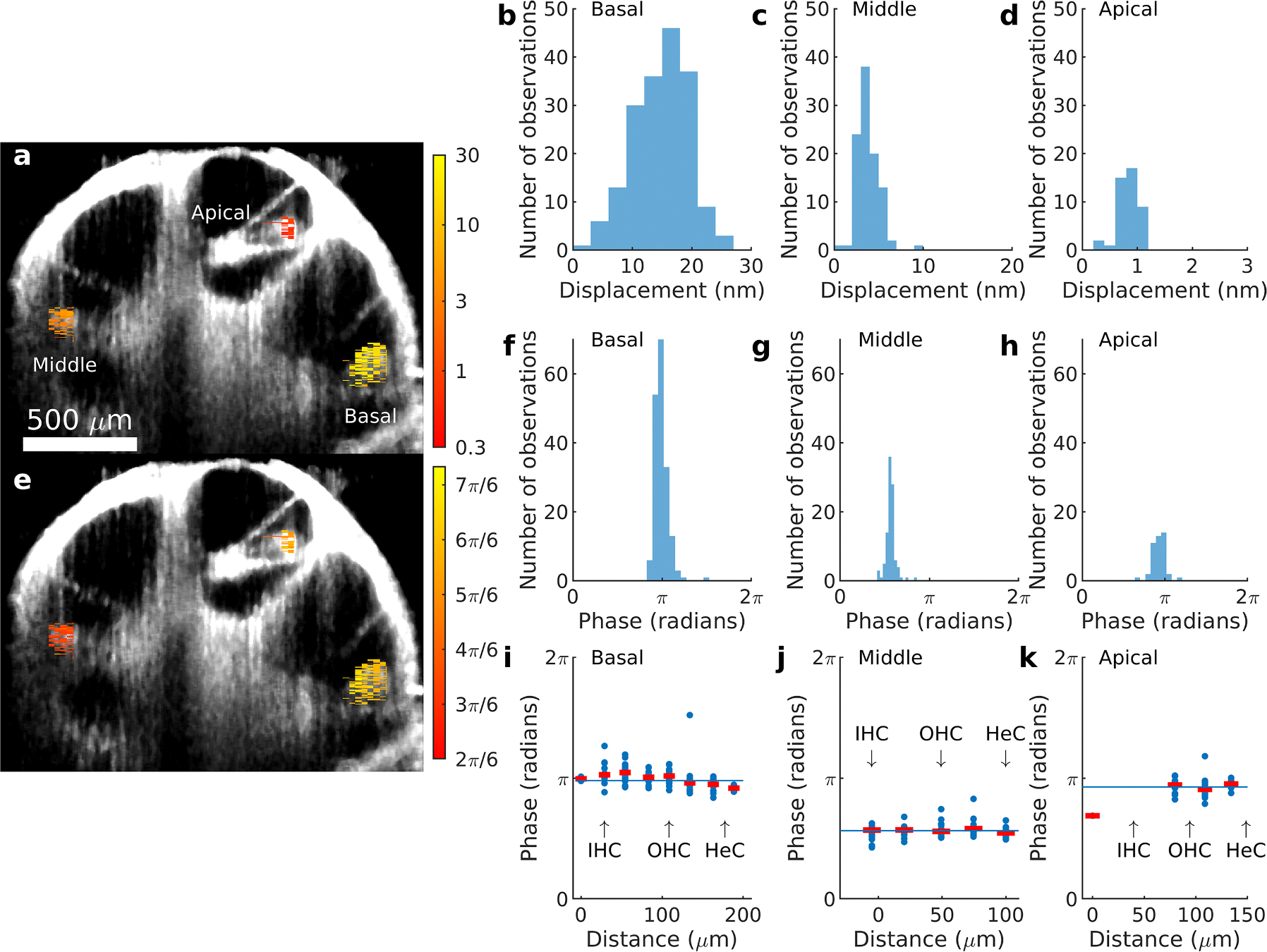
Distribution of phase and amplitude values measured from the organ of Corti. **a**. The grayscale image is from a morphological scan across the apex of the guinea pig cochlea. The colored regions represent displacement magnitudes in nanometers, as given by the color scale on the right. The three standard measurement locations used in this study are indicated on the image. **b - d**. Histogram of displacements measured from each of the three locations in panel a. **e**. Morphological scan with superimposed phase values. Phase is encoded using the color scale to the right of the image. **f-h**. Phase histograms from the same pixels as in b-d. **i-j.** Blue dots show phase values of individual pixels at different radial positions. Red horizontal bars show the median phase value at each radial position. Thin blue line is the median value of all pixels in the plot. The approximate locations of the inner hair cells (IHC); outer hair cells (OHC) and Hensen cells (HeC) are indicated. Stimulus level in all panels of this figure was 34 dB SPL (multitone stimulus, the stimulus level 34 dB SPL refers to the level of each stimulus component). All histograms plot data at 134 Hz, the best frequency of the basal location.

After positioning the mirror, the scanhead of a Thorlabs Telesto III optical coherence tomography system was positioned above the mirror, and the position of the animal’s head adjusted until a cross-sectional image of the cochlea similar to the one in Fig. 1a was obtained. The OCT system was used for morphological imaging and recording sound-evoked displacement of the organ of Corti.

Sound-evoked displacements were recorded at several sound pressure levels, before and at approximately 10-minute intervals after giving the animal an intraperitoneal injection of the diuretic furosemide, which reduces the endocochlear potential (*e.g.* Ruggero and Rich, 1991). When the endocochlear potential is reduced, the force driving ions through mechanically sensitive ion channels decreases, leading to a decreased magnitude of hair cell receptor potentials. This depresses active force generation by the outer hair cells, transitioning the cochlea to a “passive” state where sound-evoked vibration amplitudes are determined by the mechanical properties of the cochlear partition and not by forces generated by outer hair cells.

Recordings were usually complete within 4 hours. Following the recordings of sound-evoked displacements from the organ of Corti, the animal was euthanized and positioned for recording of the stapes displacement in response to sound.

### 2.1. Data acquisition and stimulus generation

The OCT system was controlled by custom-written Labview software. To precisely synchronize stimulus generation and vibration measurements, the pixel clock of the OCT system was used as the external clock for a National Instruments PCI-6259 analog-to-digital (A/D) converter. Each A-line of the OCT image therefore corresponds to one sample of the sound waveform. The analog output from the A/D board was routed to the loudspeaker positioned in the ear canal. The A/D board was also used to sample the signal from the round window electrode and from the Knowles microphone mentioned above.

Data were acquired at 10 kHz sample rate. For each position included in the vibration scan, OCT spectra were acquired for 1 second and the raw spectra stored on disk for further processing. In a typical experiment, 50–70 points were scanned across the apex, resulting in a lateral distance between the scan points ranging between 20 and 30 μm. According to the manufacturer, the distance between pixels in the Z direction is 3.49 μm, and the Z axis resolution approximately 4.2 μm in water. In a biological experiment, tissue scattering as well as variations in refractive index along the beam path will affect this value.

The acoustic stimulus was a tone complex with 15 approximately logarithmically spaced frequency components spanning the range from 60 to 1000 Hz. The frequency of each tone within the complex was adjusted to ensure that an integer number of cycles was present within the acquisition window. Stimulus levels were systematically altered over the range 34 to 54 dB SPL per frequency component. All frequency components in the complex were presented at equal sound pressure. The OCT system saved the raw spectra to disk. To avoid transients, the tone complex had 5-ms rise and fall time.

### 2.2. Signal processing

The acquired data were processed using Matlab (R2024a; The Mathworks Inc). Briefly, each M-scan of 10 000 spectra was Fourier-transformed after subtracting the source spectrum. The amplitude of this Fourier transform is the morphological image, which was used to identify pixels inside the organ of Corti. The phase values are used to compute displacements using a second Fourier transform, as described by Wang and Nuttall (2010).

In the experiments reported here, three different regions of the organ of Corti were selected, located approximately 360, 2060, and 3900 μm from the helicotrema. Measurements of the length of the guinea pig basilar membrane give somewhat varying values (18.5 mm, Lee et al 2010; 20.3 mm, Viberg and Canlon 2004; 20.8 mm, Hofman et al 2009). Using the mean of these values (19.9 mm), our measurements thus span the most apical 20 % of the cochlea. Note that previous measurements (Recio-Spinoso et al 2023) only included one site within the cochlear region that we examined, which limits the possibilities for direct comparisons between datasets.

Using the morphological OCT images, regions of interest were drawn covering each of the three organ of Corti sites that were visible. The region of interest did not include the basilar membrane. For every pixel within the region of interest, the signal-to-noise ratio was computed by dividing the amplitude at 200 Hz with the mean amplitude of surrounding bins in the amplitude spectrum. Pixels where the sound-evoked displacement was more than 10 standard deviations above the noise floor were included in subsequent analyses.

The median phase value of pixels with adequate signal-to-noise ratio was used to compute the group delay. Group delays were calculated by first subtracting the phase of the stapes displacement, unwrapping the data, and then fitting the resulting phase curves with a fourth-order polynomial, the derivative of which was determined analytically. Dividing the negative of the derivative with 2π yields the group delay in seconds. While delays could theoretically be computed directly from phase differences among the sites, such calculations are unreliable for dispersive systems, of which the cochlea is one. We also computed group delays using simple as well as central differences. The results were similar, except that these methods gave much increased noise. All phase data are shown after subtraction of the stapes phase.

### 2.3. Phase precision measurements

To our knowledge, the phase precision of OCT vibrometry systems has not been rigorously assessed. This issue is important, because group delays are calculated using the derivative of the measured phase; derivatives tend to inflate noise by emphasizing minor differences across data points, making group delays highly sensitive to phase noise. To evaluate whether this error is significant, an artificial target was constructed from a piece of boxing wax (Kerr Manufacturing Company) with a 0.17-mm thick microscope cover slide on top. The upper and lower surface of the cover slip had reflectivity similar to the thin cochlear bone at the apex while the red wax’s reflectivity was in the same range as the organ of Corti. The target was mounted on a piezoelectric stack that made it possible to compare the measured vibration phase values to the known input to the piezo stack. The data were processed using the same techniques as when processing cochlear vibration data. Statistical analysis was performed in Matlab and in R.

## 3. Results

The grayscale image in Fig. 1a shows a morphological OCT image acquired from the apex of the cochlea in a living, anesthetized guinea pig. In a typical experiment three different sites were visualized, at approximate distances of 360, 2060 and 3900 μm from the helicotrema. Note that all three locations are within the apical 20 % of the cochlea, hence the term “basal” in Fig. 1a and in the text below refers to the measurement location 3900 μm from the helicotrema (and not to some location at the base of the cochlea).

Displacements evoked by an acoustic stimulus at 34 dB SPL and 134 Hz, the best frequency of the basal location, are superimposed on the morphological scan, the color scale on the right giving the displacement magnitude in nanometers. Displacements varied more between measurement sites than they did within them, as evidenced by the similarity in color for each site. This is more accurately displayed in the histograms in Fig. 1b–d. Here it is evident that the largest number of pixels with good signal-to-noise ratio was found at the basal site (Fig. 1b), with displacement magnitudes decreasing for the middle (Fig. 1c; note the different scale on the x axis) and apical sites (Fig. 1d). The data in Fig. 1b–d are consistent with a normal distribution (Kolmogorov-Smirnoff test, all p values *>* 0.23).

Fig. 1e provides a color-coded display for the corresponding phase data. After unwrapping, the phase values were consistent at each site, as evident from the similarity of colors in Fig. 1e and the narrow histograms in Fig. 1f–h. The histograms in Fig. 1f and g are not consistent with a normal distribution (Kolmogorov-Smirnoff test, p = 0.03 in both cases). Although the phase data from the apical location were not significantly different from the normal distribution, the relatively small number of pixels that passed the signal-to-noise criterion at this location limits the power of the test. Given the non-normal distribution of most phase data, medians and interquartile ranges are more appropriate than means and standard deviations for descriptive statistics and calculations of group delays.

Fig. 1i shows the phase of the 181 pixels at the basal location that passed the signal-to-noise criterion, plotted as a function of radial position (red horizontal bars show the median phase for each position). Note that the inner hair cell region had a slight phase lead over the outer hair cells and Hensen cells. The maximal difference between the median values was 0.41 radians (23°; p=5.5×10^−19^; Kruskal-Wallis test). Since the outer hair cell region had many pixels that passed the signal-to noise criterion, the median phase value of all basal pixels (thin blue line in Fig. 1i) is close to that of the outer hair cells. Data from the middle and apical locations (Fig. 1j and k) do not show significant differences between radial positions (p values of 0.11 and 0.2, respectively, Kruskal-Wallis test); the median of all phases was close to the outer hair cell region’s phase (thin blue line). We conclude that the averaging used by Burwood et al results in group delays that primarily reflect the motion of the outer hair cell region.

**Fig. 2.**
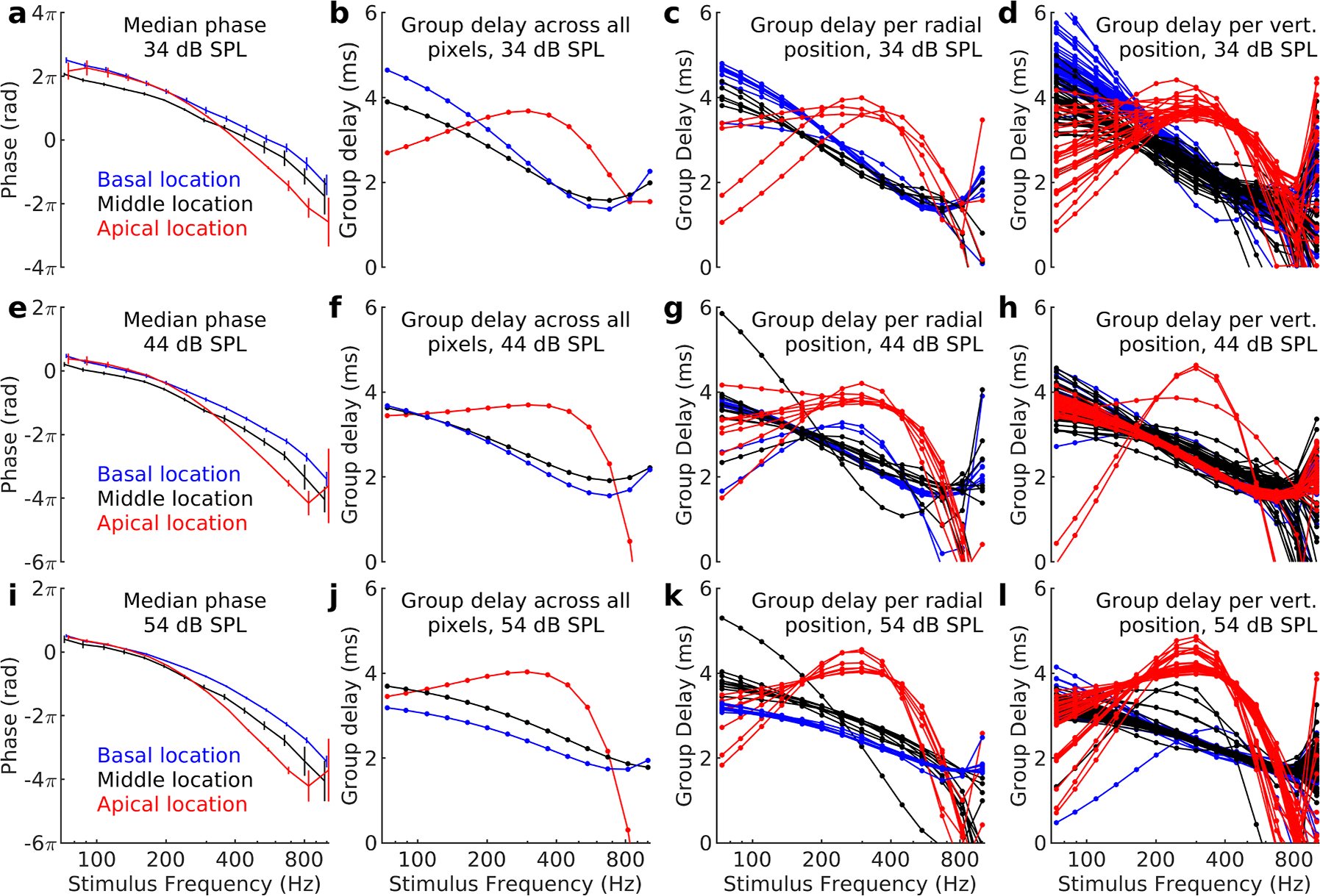
Position-specific group delays are similar to the overall group delay. **a,e,i.** Phase values calculated by taking the median phase of every pixel that passed the signal-to-noise criterion. The phase of the stapes vibration was subtracted before unwrapping the data. **b,f,j.** Group delays calculated from the median phase values in panels a, e, and f. **c,g,k.** Group delays calculated from the median phase at each radial scan position. **d, h, l.** Group delays calculated from the median phase at each vertical scan position. Stimulus levels are indicated on each graph. The same color scheme is used in all panels.

Do these radial phase differences influence the group delay? To address this question, Fig. 2 plots phase values and group delays from different radial and vertical positions in a representative preparation, together with group delays computed from the median of all pixels with good signal-to-noise ratio.

The first row of plots (Fig. 2a–d) show data acquired at 34 dB SPL. In Fig. 2a, median phase curves from the basal (blue), middle (black) and apical (red) locations are shown together with the interquartile ranges (vertical bars). As expected from the narrow histograms in Fig. 1, the interquartile ranges are quite small, except at frequencies well above the best frequency. The slope of the phase curves from the basal and middle locations are similar, whereas the apical location has smaller slope than the other two curves at low frequencies and an increasing slope for frequencies above 300 Hz.

To determine group delays, each unwrapped phase curve was fitted with a fourth-order polynomial, the derivative of which was determined analytically. The derivatives were then used to compute the group delays shown in Fig. 2b. As expected from its smaller slope, the apical site had the shortest group delay at low frequencies, followed by the middle and the basal site. As the stimulus frequency increased, the group delays of the basal and middle locations became more similar, whereas the apical site showed a more complicated pattern with an initially increasing group delay followed by decrease at frequencies above 400 Hz. To ascertain that these results were not an artefact of the computation method, derivatives were also computed by simple as well as central differences. These numerical methods resulted in group delays with increased noise, but the pattern in Fig. 2b remained.

The phase curves and group delays shown in Fig. 2a and b were computed by taking the median value of all pixels with adequate signal-to-noise ratio. If there are systematic variations in phase across the width of the organ of Corti or across pixels acquired at different depth, the result could be an averaged phase pattern that does not truly reflect organ of Corti motion, as suggested by Recio-Spinoso et al (2023). To address this concern, group delays were computed for each radial position across the organ of Corti.

In Fig. 2c, note that group delays from the middle location (black lines) show only minor variations. All the curves recorded from this location are close to overlapping each other, suggesting an absence of radial variations in group delay. A similar picture is evident for the basal location (blue lines). Of the 8 radial positions that were measured from this site, 7 had longer group delay than the middle site at frequencies below 300 Hz. At the most apical position, motion was recorded at four radial positions (red lines in Fig. 2c). At low stimulus frequencies, this measurement site had the shortest group delay, but at frequencies between 250 Hz and 550 Hz, it had the longest group delay. Although low-frequency group delays were variable at the apical site, all of the recorded curves show shorter delay than the basal and middle locations.

What about phase differences across the vertical dimension of Fig. 1e? Since the resolution along the vertical axis is higher than the radial one, a larger number of phase curves are shown in Fig. 2d. At the lowest stimulus frequency (74 Hz), the basal site had longer group delay than the middle site (Mann-Whitney U test, p = 5.7×10^−13^), which had longer group delay than the apical location (Mann-Whitney U test, p = 8.7×10^−8^). Thus, the pattern shown in Fig. 2b is confirmed. At higher stimulus frequencies, the basal and middle site had similar group delay, but the longest delay occurred at the apical site for frequencies between 250 and 550 Hz. Since neither radial nor vertical averaging of group delays affected the result in Fig. 2b, we conclude that the unusual group delay that we describe are not a consequence of averaging pixels from different organ of Corti locations.

The low-frequency group delays in Fig. 2b range between 2.7 and 4.6 ms. This substantial delay is consistent with energy being carried by traveling waves to the apex. However, at 34 dB SPL the most apical location has the shortest delay at low frequencies, while the middle site has shorter delay than the basal one through a large part of the response range. These features are not consistent with a conventional traveling wave.

The middle row of curves in Fig. 2 (panels e-h) show data acquired at 44 dB SPL in the same preparation as panels a-d. Note that the phase curves from the basal and middle locations in panel e have close to identical slope through most of the frequency range. The phase curve from the apical location is very similar to the basal location at low frequencies, but the slope increases substantially above 200 Hz. As expected from the similarity of the phase curves, group delays were similar for the basal and middle locations throughout the frequency range tested here. The pattern for the apical location is similar to the one in Fig. 2b, with a low-frequency segment with short group delay, followed by increase and then rapid decrease. These patterns did not depend on either radial or vertical position, as seen in panels 2g and h. Group delays for the middle location (black in Fig. 2g) are similar to the basal one throughout the frequency range (blue in Fig. 2g). Both the middle and the basal location have two outliers, but neither outlier significantly affects the overall group delay. Fig. 2h shows that averaging along the other direction does not fundamentally alter the result; group delays overlap for most frequencies at the basal and middle locations, with a more complicated pattern being evident at the most apical site.

Further increase of the stimulus level to 54 dB SPL led to some differences among the curves. The largest change occurred for the basal location, which now had the shortest group delay, followed by the middle and then the apical location. One hallmark of a traditional traveling wave is that the group delay increases at more apical sites. Data at 54 dB SPL are the only ones that are reasonably consistent with this assumption, suggesting that traveling wave motion is a feature of the passive mechanics of the apical organ of Corti, but conventional traveling waves do not necessarily occur at lower stimulus levels, where amplification provided by the outer hair cells strongly influence sound- evoked responses.

If we compare the group delay curves at 34, 44 and 54 dB SPL (Fig. 2b, f, and j), it becomes evident that the middle and apical locations had group delays that were nearly invariant with level. For the middle location, there are minor changes to the group delay curves as the level increases. For the apical location, group delay curves change with level at very low frequencies but are largely independent of stimulus level at frequencies above 150 Hz. The basal location however shows pronounced level dependence, with decreasing group delay as the level increases.

**Fig. 3.**
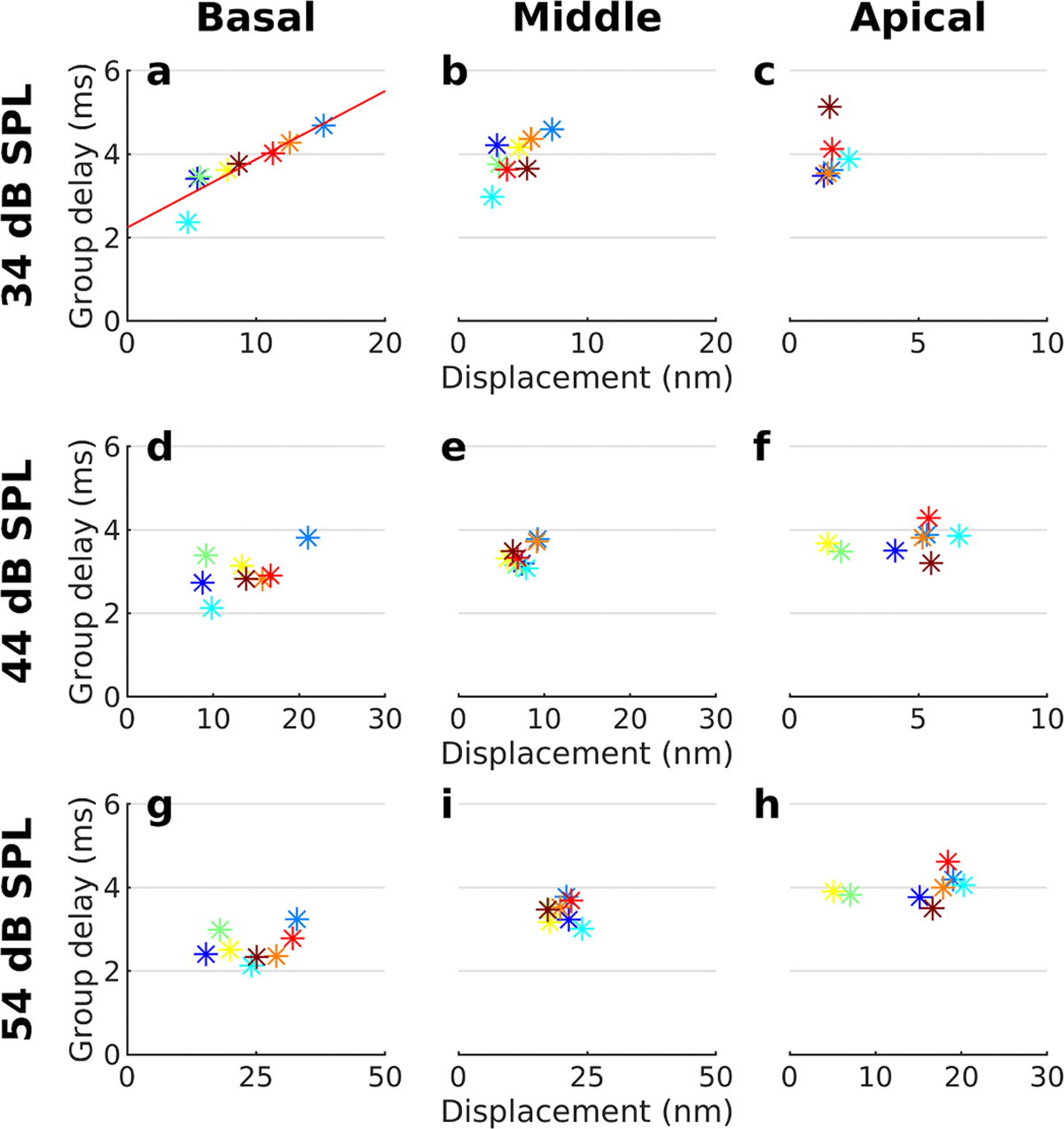
Relation between peak group delay and peak displacement. **a.** At the most basal measurement location, a linear relation between sound-evoked displacement and the peak group delay was evident, but only at 34 dB SPL. The red line was drawn by linear regression, Group delay = 1.64×10^−4^ × displacement + 2.24 ms (p=0.002; 34 dB SPL). The median peak displacement was found at 150 Hz for the basal location, 74 Hz for the middle one, and 330 Hz for the apical location. **b,c.** Corresponding data for the middle and apical locations. **d-h.** Relation between displacement and group delay at 44 and 54 dB SPL.

On examining data from our sample of 8 animals with normal hearing, it became evident that group delays varied depending on the sensitivity of the individual preparation. To highlight this, Fig. 3 shows how the peak group delays depend on the peak displacement of the organ of Corti. Note that peak displacement was measured at the best frequency of each site. Since the apical site generally had higher best frequencies than the other sites, its group delay reflects the peak seen in Fig. 2b.

For the basal location, preparations with larger displacements had longer group delays, as evidenced by the regression line across the data points (Fig. 3a; p=0.002; r^2^=0.82; analysis of variance). For the middle location, a regression line across the points had steeper slope but failed to reach statistical significance (p=0.06; r^2^=0.46). For the most apical location, displacements were smaller and did not vary much between preparations, even though the group delays varied.

Although data at 44 dB SPL are suggestive of a linear relation between displacement and group delay at the most basal measurement location, the regression lines were not significant for any of the three locations at this stimulus level.

Only at 54 dB SPL are the data consistent with traditional models of cochlear function, since at this stimulus level the group delays at the best frequency of each location are shortest for the most basal location, followed by the middle and apical locations.

**Fig. 4.**
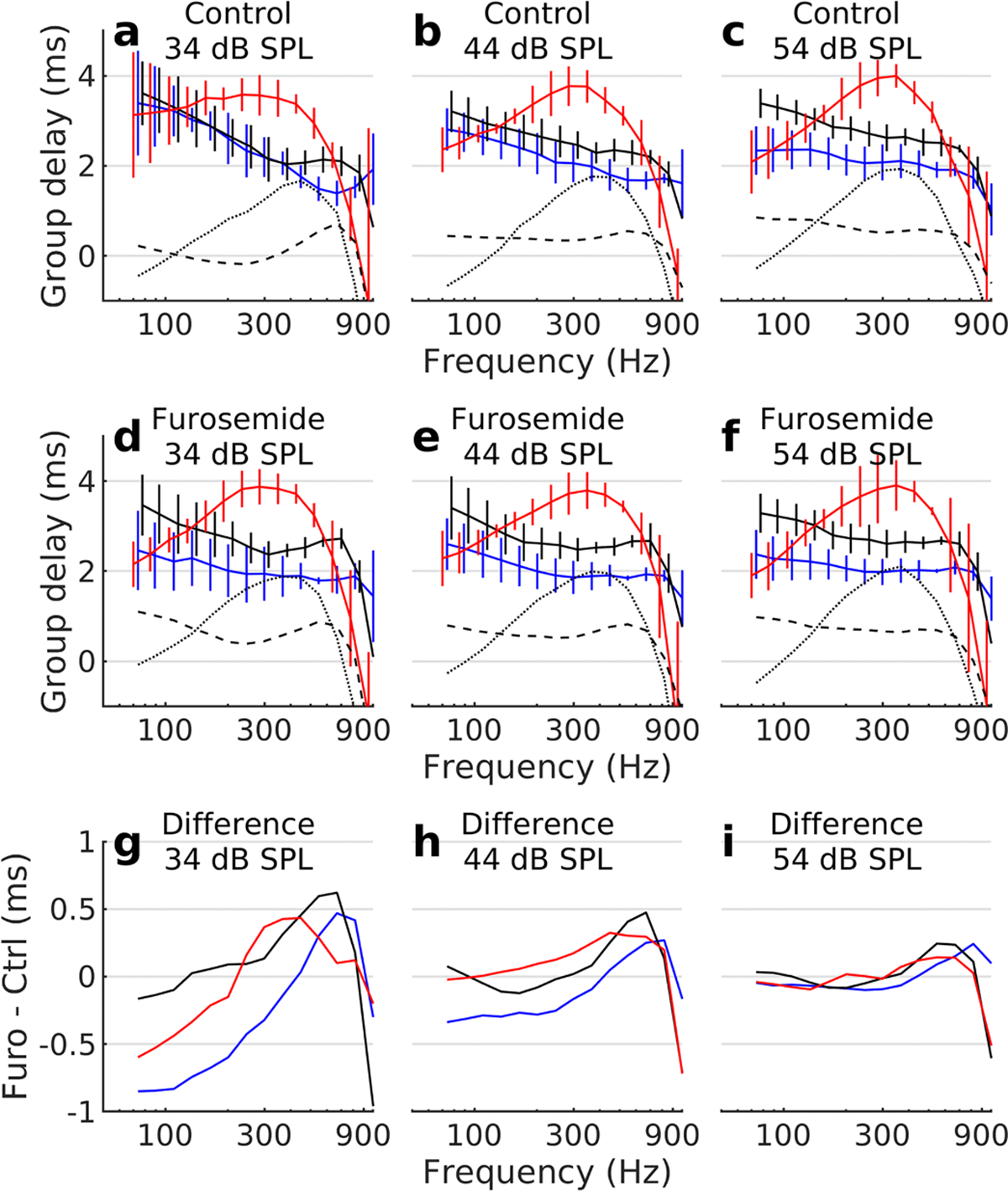
Outer hair cell force production affects group delays. Median group delays and their interquartile ranges across 7 preparations before (a-c) and 90 minutes after (d-f) intraperitoneal furosemide. In panels a-f, dashed black lines show difference in group delay between the middle and the basal site; dotted lines are the difference in group delay between the apical and the basalmost site. The difference between group delays measured at peak furosemide (furo) effect and group delays measured before furosemide is shown in panels g-i. In all panels of this figure, red color marks the apical site, solid black lines mark the middle site, and blue lines show data from the basal location. Vertical bars denote the interquartile range.

Since the data in Fig. 3 are at different frequencies for each site, we present in Fig. 4 group delay curves averaged over our sample of 8 preparations. At 34 dB SPL (Fig. 4a), group delays for the most basal and the middle location largely overlapped. As a result, the median difference in group delay between these sites is zero or negative through much of the frequency range (dashed line in Fig. 4a). As the stimulus level increased, delays between the middle and the basal site gradually developed (Fig. 4b and c, dashed lines). At 44 dB SPL, the median difference in group delay between the basal and middle sites was 0.4 ms; this difference increased to 0.57 ms at 54 db SPL. The most apical location behaved differently. It consistently had the shortest group delay at low frequencies (dotted lines in Fig. 4a–c), but the longest group delay near its peak response region. A slight increase of group delay occurred with increasing stimulus level.

Furosemide changed these patterns substantially. At peak furosemide effect, most of the level-dependent change in group delays had vanished (Fig. 4d–f), and the basal site now had shorter group delay than the middle site at all stimulus levels. The most apical site however remained an outlier, with short group delay at very low frequencies, and a peak near 300 Hz (the best frequency of that location).

To further highlight the change induced by furosemide, Fig. 4g–h shows the difference in group delays before and after furosemide. For the basal site, furosemide caused group delays to become shorter at frequencies below 500 Hz at 34 dB SPL (Fig. 4g, blue curve). At frequencies higher than this, group delays increased. For the middle site (red lines in Fig. 4g), group delays also became shorter after furosemide, but only at frequencies below 250 Hz. The apical site showed increased group delay through most of its response range. Changes induced by furosemide became smaller at higher stimulus levels (Fig. 4h and i).

The lack of group delay differences between the middle and the basal site (Fig. 4a) and the shorter group delay at low frequencies at the most apical site shows that traditional traveling waves are not present within the apex. Their presence in other cochlear regions is however not disputed.

Since the above data shows a pattern at odds with traditional models of cochlear function, it is fair to ask whether technical factors may contribute. To our knowledge, there is little published information about phase measurements with OCT vibrometry, which makes it important to examine the precision of the technique for measuring small phase differences among vibrating objects.

**Fig. 5.**
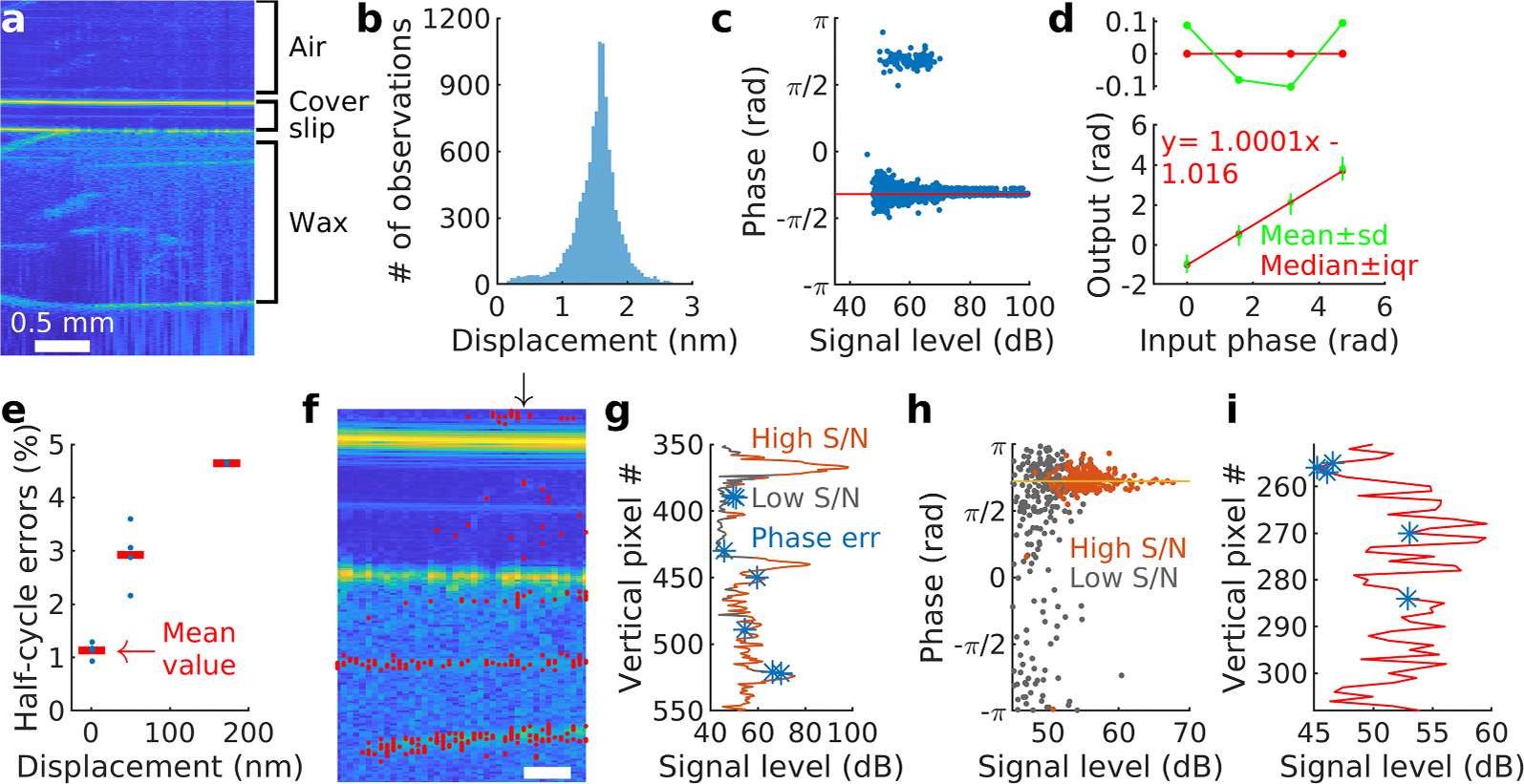
Phase measurements on an artificial target reveal bimodal distribution of phase values. **a.** Structural OCT image of the target, which was mounted on top of a stack of piezoelectric elements. **b.** Distribution of amplitude values. Stimulation frequency, 200 Hz. **c.** The bimodal distribution of phase values become apparent when the phase of individual pixels is plotted versus signal level (amplitude data are shown in panel b; median displacement of 1.6 nm). **d.** Lower graph shows the measured phase as a function of the input phase. The displacement magnitude was 50.5 ± 8 nm (median ± interquartile range). Top graph shows the residuals for the linear fit when using the median values (red, r^2^ = 1; root mean squared error 0.00012 nm) and the mean (green; r^2^=0.996; rms error 0.13 nm). **e.** The fraction of pixels with half-cycle errors increases with the magnitude of motion. Only pixels that passed the signal-to-noise criterion were included in this analysis. **f.** Pixels with phase errors are not randomly distributed. Red dots are pixels with phase errors, here superimposed on a B-scan of the target. Scale bar, 0.25 mm. The vertical arrow shows the A-line plotted in panel g. **g.** A single A-line through the artificial target. Pixels with good signal-to-noise ratio (S/N) are plotted in orange; pixels not fulfilling the S/N criterion in light gray. Pixels with erroneous phase are marked with blue asterisks. **h**. Effect of the S/N criterion on the measured phase of organ of Corti sound-evoked displacements. Data are from the same preparation as in Fig. 1a. Gray dots show pixels that did not pass the mechanical S/N criterion, orange dots are pixels whose displacement at the stimulus frequency was more than 10 standard deviations above the mean value of surrounding bins in the spectrum. The median displacement of high S/N pixels was 14.8 nm; the low S/N data had 9.4 nm median displacement. **i**. The red curve is a single A-line through the outer hair cell region on the same preparation as in panel h. Pixels with anomalous phase values are indicated by blue asterisks.

To determine the phase consistency of OCT vibrometry, we constructed an artificial target by placing a glass cover slip on a piece of red wax, mounting the assembly on a high-performance piezoelectric actuator. The peak reflectivity of the cover slip was approximately 97 dB, whereas the bone at the apex of the cochlea had typical peak reflectivity around 90 dB. The peak reflectivity of the organ of Corti and the red wax were both approximately 75 dB. Hence, the cover slip has higher reflectivity than the bone, whereas the red wax and the organ of Corti have similar reflectivity. Fig. 5a shows an OCT image of this target, with the different parts marked on the right of the image.

When examining the vibrations of this target, we used the same principle as when measuring cochlear vibrations. A region of interest covering the cover slip and wax was drawn, and pixels with adequate signal-to-noise ratio extracted. Mimicking the real measurements, pixels were included if the peak at the stimulus frequency (200 Hz) was larger than 10 standard deviations from the mean value of the surrounding frequency bins. Fig. 5b shows that displacements of this target formed a sharp peak centered on 1.5 nm. Plotting the corresponding phase of the displacement as a function of the reflectivity of each pixel revealed an interesting pattern. While 99 % of all pixels show phase values that scatter around the median value (−1.01 radians, red horizontal line in Fig. 5c), about 1 % of all pixels are off by half a cycle (180°). This bimodal distribution reinforces the conclusion from the statistical analysis of phase histograms presented in Fig. 1. It is inappropriate to use means and standard deviations when describing or processing phase data from this commercially available OCT system.

To illustrate the effects of such phase errors, we plot data from four separate acquisitions in Fig. 5d. The phase of the voltage driving the piezo stack was varied over a 270-degree range, while measuring the vibration phase with OCT as described above. The red dots show phase values computed by taking the median of all pixels that fulfilled the signal-to-noise criterion. The interquartile range was very narrow (0.0064 to 0.0068 radians, 0.37° to 0.39°), making the red vertical bars nearly invisible. By contrast, standard deviations were about 86 times larger than the interquartile range, as illustrated by the green dots and bars. The regression line drawn from the median values has slope very close to 1, the adjusted coefficient of determination was 1, and the fit statistically significant (p=1.2×10^−9^; anova). The top graph in Fig. 5d shows the residuals of the fit. Residuals were near zero when the fit was based on median phase values (red) but increased drastically when the mean phase values were used for fitting (green; the adjusted coefficient of determination was 0.996; p=0.0014). Thus, OCT can measure the phase of nanometer displacements with extreme precision, and the precision is considerably improved if median phase values are used.

It may appear that errors shown in Fig. 5d are small regardless of the method for averaging. However, since calculation of group delays require the derivative of the phase across frequencies, small errors will be amplified. The effects that this has on the group delay depends on the method for computing the derivatives. If simple or central differences are used, the effect is larger than with the polynomial fitting method used in Fig. 2.

To determine whether the precision of phase measurements is affected by the amplitude of displacement, measurements were repeated at displacements ranging from 1.6 to 172 nm. An increasing proportion of pixels showed half-cycle errors when the displacement increased, an effect that was statistically significant (Fig. 5e; one-way anova, p=5.6×10^−5^, F=54 over 9 degrees of freedom; note that we only considered pixels that passed the strict mechanical signal-to-noise criterion described under methods).

Are pixels with phase errors randomly distributed across the OCT image? To answer this question, Fig. 5f shows pixels with phase errors (red dots) superimposed on the morphological scan. It is evident that pixels with phase errors tend be located near local signal peaks, such as the faint horizontal green line near the bottom of the image and the top surface of the cover slip. This is further highlighted in Fig. 5g, which shows a single A-line from the same acquisition as in panel f. Here, pixels that fulfilled the signal-to-noise criterion are orange, and pixels with phase errors marked by asterisks (note that we did not examine the phase of pixels that did not fulfill the signal-to-noise criterion). Most, but not all, pixels with phase errors occur close to local peaks in reflectivity.

We also examined the effect of the signal-to-noise criterion on the accuracy of the measured phase. A total of 459 pixels were present within the region of interest corresponding to the basal measurement site in Fig. 1a (stimulus level, 34 dB SPL), 181 of which fulfilled the signal-to-noise criterion (Fig. 5h, high S/N pixels in orange). Among those 181 pixels, there were two outliers at phase values of 0.49 and −3.11 radians, but the remainder clustered near the median value of 2.27 radians (interquartile range, 0.26 rad). The data in Fig. 5h suggest a different criterion could be effective. If pixels with signal level in the morphological image better than 60 dB are selected (19 pixels fulfill this criterion), their median phase value is nearly identical to the median value of the larger group of 181 pixels. Pixels fulfilling neither criterion used above had median value 2.05 radians, with an interquartile range of 1.66 radians (Fig. 5h, low S/N, gray).

In the organ of Corti data, few pixels had phase errors when the standard signal-to-noise criterion was used. By removing the criterion, more pixels become available for analysis, allowing their distribution to be examined. Fig. 5i plots a single A-line containing 58 pixels from the outer hair cell region. From these 58 pixels, 53 clustered near a phase value of 2.3 radians. The remaining 5 pixels, with median phase −2.8 radians, are marked with blue asterisks on the plot. While three of those are in a region of low reflectivity where good vibration data cannot be expected, two pixels with deviant phase were found in regions with good reflectivity. Both are located near local signal level peaks, suggesting that the signal leakage mechanism demonstrated for OCT amplitude data (Lin et al 2017) may be relevant.

In conclusion, it is essential to select pixels having either a good signal-to-noise ratio or a high signal level in morphological images to obtain reliable phase values when measuring organ of Corti displacements with OCT.

## 4. Discussion

With the introduction of OCT, it became possible to study apical cochlear mechanics without the artefacts that affected previous studies. The new studies show that the apex of the cochlea does not function as predicted by well-established theory (Warren et al 2016; Burwood et al 2022). In particular, Fig. 2 in the present paper shows that the most basal measurement site has longer group delay than the middle or apical measurement location at 34 dB SPL, a result inconsistent with conventional understanding of cochlear traveling waves. It is however important to state that these results do not exclude traveling waves in other parts of the cochlea; the phase data in Fig. 2 are consistent with a wave traveling through more basal locations before reaching the apex, but at low stimulus levels, a conventional traveling wave does not appear to be present within the apex.

An absorptive nonlinear system, such as the cochlea, can be characterized by several types of propagation delays. The phase delay is obtained by expressing the phase at some frequency in units of time, usually using a pure-tone stimulus. This can be used to describe a linear system but is of less interest here. More interesting is the signal-front delay, which describes the latency of the impulse response (Ruggero and Temchin 2007). There is no straightforward way of determining the signal-front delay from the response to tones or tone complexes, but such stimuli allow determination of the group delay, which quantifies the frequency-dependent delay of energy transport. Of these three quantities, the group delay is thought to be most closely related to the cochlear active process (Ruggero and Temchin 2007). This view is supported by our data, since Fig. 4 shows that furosemide caused a substantial level-dependent shortening of the group delay for the basal and middle measurement location.

Our previous publication (Burwood et al 2022) was questioned on the grounds that averaging vibrations across the width of the organ of Corti could produce phase relations that do not reflect the “true” phase. The present analyses were performed to allay such concerns. While there are minor variations in phase across the organ of Corti at the basal measurement site (Fig. 1i), but not at the middle or apical site (Fig. 1j. and k), these minor phase changes lack substantial influence on the computed group delay: As shown in Fig. 2, group delays are nearly identical across different radial locations for both the basal and apical measurement site (Fig. 2c). Looking at different vertical positions (Fig. 2d) does not change the picture. The group delays still cluster around the median value shown in Fig. 2b. The most problematic site is the apical one, because low response amplitudes and smaller organ of Corti cross-sectional area at this location means that fewer pixels pass the signal-to-noise criterion. Consequently, there is larger variability in the group delays from this location. Despite the variability, most group delay curves from the apical location show shorter group delay at very low frequencies than the other locations, but closer to the best frequency, the apical location has the longest group delay (Fig. 4).

Since direct measurement of stereocilia motion are not feasible in living animals (but see Hakizimana and Fridberger 2021), it is as yet unclear how these group delays translate into auditory nerve excitation. Auditory nerve tuning curves from the apex vary between bandpass, lowpass, and complex shapes (*e.g.* Versteegh et al 2011, Fig. 3a), suggesting complex interactions between the tectorial membrane, stereocilia, and organ of Corti motion.

Fig. 5 shows that the unexpected phase results are not caused by technical factors. Although half-cycle errors are present and become more prevalent at larger displacement, their effect is nullified by the use of medians rather than means. Since many pixels with phase errors are located near structures with high reflectivity (Fig. 5f, g and i), the mechanism that generates them may be the signal leakage demonstrated for amplitude data by Lin et al (2017).

The overall result of these new analyses and measurements is that the concerns about averaging of pixel values across different parts of the organ of Corti (Recio-Spinoso et al 2023) do not apply to our data.

While the calculation of group delays may appear straightforward, we note that it involves numerical derivatives, which have a tendency of introducing significant noise. This source of noise is minimized if derivatives are computed from polynomial fits of unwrapped phase data, rather than by central or simple differences. Unfortunately, previously published work on apical group delays contains no information about calculation methods (Recio-Spinoso and Oghalai, 2018). Furthermore, from the three locations studied by Recio-Spinoso et al (2023) only one was within the region that we examined here and elsewhere (Burwood et al 2022).

Having laid concerns about averaging and technical factors to rest, we ask what the mechanism underlying the unexpected apical motion pattern may be?

In their study of the morphology of the guinea pig cochlea, Yarin et al (2014) showed that the width of the organ of Corti increases from the base toward the apex. This tonotopic relation was described previously and is not surprising. However, starting at the 750-Hz place, the width of the organ of Corti is constant until the 100-Hz place is reached, at which point the width starts decreasing. This suggests that the mechanical properties of the organ of Corti may be similar between the 100-Hz and the 750-Hz place, which may contribute to the apparent similarity in best frequency and lack of apical traveling wave motion that we observe. The smaller width close to the helicotrema may then contribute to the higher best frequency we observed; it could also serve to reduce back-reflections caused by the helicotrema impedance discontinuity.

Local discontinuities in organ of Corti mechanics and morphology are present in certain other mammals (Russell and Kössl, 1999; Manley et al, 2024) and generally reflect different ecological niches. Niche specializations may also be present in other species, including what is observed here in the guinea pig and plausibly, although currently unidentified, in humans.

## Data Availability

Data will be made available on request.
